# Antimicrobial and Antioxidative Activity of Newly Synthesized Peptides Absorbed into Bacterial Cellulose Carrier against *Acne vulgaris*

**DOI:** 10.3390/ijms22147466

**Published:** 2021-07-12

**Authors:** Iwona Golonka, Katarzyna E. Greber, Monika Oleksy-Wawrzyniak, Justyna Paleczny, Andrzej Dryś, Adam Junka, Wiesław Sawicki, Witold Musiał

**Affiliations:** 1 Department of Physical Chemistry and Biophysics, Wroclaw Medical University, Borowska 211A, 50-556 Wroclaw, Poland; iwona.golonka@umed.wroc.pl (I.G.); andrzej.drys@umed.wroc.pl (A.D.); 2 Department of Physical Chemistry, Faculty of Pharmacy, Medical University of Gdańsk, Al. Gen. J. Hallera 107, 80-416 Gdańsk, Poland; katarzyna.greber@gumed.edu.pl (K.E.G.); wieslaw.sawicki@gumed.edu.pl (W.S.); 3 Department of Pharmaceutical Microbiology and Parasitology, Wroclaw Medical University, Borowska 211A, 50-556 Wroclaw, Poland; monika.oleksy-wawrzyniak@umed.wroc.pl (M.O.-W.); justyna.paleczny@student.umed.wroc.pl (J.P.); adam.junka@umed.wroc.pl (A.J.)

**Keywords:** peptides, antimicrobial activity, *Acne vulgaris*, antioxidant properties, photosensitivity, bacterial cellulose

## Abstract

The ongoing search for effective treatment of Acne vulgaris is concentrated, i.a., on natural peptides with antimicrobial properties. The aim of this work was the development of new amino acid derivatives with potential activity on dermal infections against selected microorganisms, including the facultative anaerobe *C. acne*. The peptides P1–P6 were synthesized via Fmoc solid phase peptide synthesis using Rink amide AM resin, analyzed by RP-HPLC-MS, FTIR, DPPH radical scavenging activity, and evaluated against *C. acne* and *S. aureus*, both deposited and non-deposited in BC. Peptides P1–P6 presented a lack of cytotoxicity, antimicrobial activity, or antioxidative properties correlated with selected structural properties. P2 and P4–P6 sorption in BC resulted in variable data, i.a., confirming the prospective topical application of these peptides in a BC carrier.

## 1. Introduction

*Acne vulgaris* belongs to dermatoses of complex origin, engaging interactions between microbiological, genetic, dietary, immunological, hormonal, and environmental factors [1]. It affects an increasing number of patients, and is diagnosed mainly within young populations; however, in adults the manifestation of acne may become acute and present with severe inflammatory lesions. One of the microbiological factors in its development is the excessive growth of the facultative anaerobe, *Cutibacterium acnes*, formerly described as *Propionibacterium acnes*, a species naturally occupying the skin’s surface and hair follicles. This leads to an increased release of pro-inflammatory compounds, resulting in immoderate activation of immune system components [2,3,4,5,6,7,8]. *C. acnes* hyaluronidase facilitates tissue decomposition and bacterial colonization, and intensifies symptoms [9,10]. The colonization of acne lesions by *Staphylococcus aureus* also strengthens inflammation. Moreover, the aforementioned bacteria exist and multiply intensively in areas already colonized or desolated by *C. acnes* [11]. At present, applied anti-acne preparations, including azelaic acid, benzoyl peroxide, salicylic acid, antibiotics, or retinoids, in numerous cases fail to succeed, leading to anxiety and reduced self-confidence; however the most important issue, here, concerns adverse drug reactions, which are often ineligible for proper documentation [12]. The ongoing search for more effective treatment is concentrated, i.a., on natural peptides with antimicrobial properties. This approach offers several advantages over conventional antibiotic therapy, including high antimicrobial efficiency against a wide range of invasive pathogens that are presently resistant to conventional antibiotics [13,14]. Natural peptides are often employed as pattern structures for the development of molecules with favorable properties for antimicrobial efficacy, stability in physiological condition, affinity and penetration capacity in microbial membranes, and economic profitability [15,16]. These peptides display favorable properties in the area of safety as they are considered biocompatible, non-irritative, non-allergenic, and non-cytotoxic to the skin [17,18]. Thus, we propose a method of replacing the missing components of the altered epidermis and stimulating the production of endogenic substances [18].

The activity of anti-acne preparations, when topically applied to the skin, may be compromised as a result of exposure to solar radiation [19], and adverse effects may be amplified due to the sensitization of skin by sunlight [20]. The application of a suitable drug carrier may prevent drug decomposition and enable controlled or targeted drug delivery into the affected skin area. Among intensively investigated modern drug carriers, one, bacterial cellulose (BC), is described as bionanocellulose and produced by *Komagateibacter xylinus* in the form of elastic, light pellicles of shape determined by the culturing vessel. The properties of BC attract attention in various industries, including textiles and dietary and electronic manufacturing [21], due to its demonstrated biocompatibility and lack of allergenicity. Thus, the application of BC as a wound dressing and in topically-used skin formulations has been gaining significant attention.

In our own studies we have found, and had confirmed by other researchers, that BC may be used for the absorption of numerous substances, while its biological activity is maintained after such procedures [22]. Some authors have proposed BC as an efficient drug carrier, presumably for controlled drug release [23]. Notably, it has already been indicated that highly hydrated BC, containing ca. 97–99% of water, acts as an alleviating agent, reducing pain and inflammatory symptoms [24]. BC has also already been used as a carrier for antimicrobial peptides; one study demonstrated BC’s suitable absorbance of peptides, while analysis of antibacterial activity has confirmed the significantly enhanced efficiency of the action of peptides released from BC [25]. It should also be noted that BC is already in application in the cosmetics industry as a facial mask for the delivery of active compounds and increased skin hydration [26].

Therefore, in this work we evaluated the activity of newly synthesized peptides de novo, or absorbed into BC, against *C. acnes* and *S. aureus*. We used the following peptides in the present work: (WK)_2_-KWK-NH_2_ (P1), (WKWK)_2_-KWKWK-NH_2_ (P2), (WR)_2_-KWR-NH_2_ (P3), (C12)_2_-KKKK-NH_2_ (P4), (KWK)_2_-KWWW-NH_2_ (P5), and (KK)_2_-KWWW-NH_2_ (P6). We hypothesized that the presence of a positively charged amino acid, i.e., lysine, in the investigated compounds may increase their affinities to the negatively charged membranes of bacterial cells, while the relatively low molecular mass of these peptides may favor their penetrability. Their antioxidant potential and resistance to solar radiation was evaluated to elucidate the practical applications of these peptides. The aim of our work was the development of new amino acid derivatives with potential activity in dermal infections against selected microorganisms, including the facultative anaerobe *C. acne*.

## 2. Results

### 2.1. Characterization of the Peptides

The purity of the newly synthesized peptides P1–P6, confirmed with RP-HPLC, exceeded 95%. MS spectra confirmed the identity of the studied compounds, as shown in Table 1. The molecular structures of the peptides are presented in Table 2.

### 2.2. FT-IR Spectroscopy of Original and Irradiated Peptides

**Figure 1:**
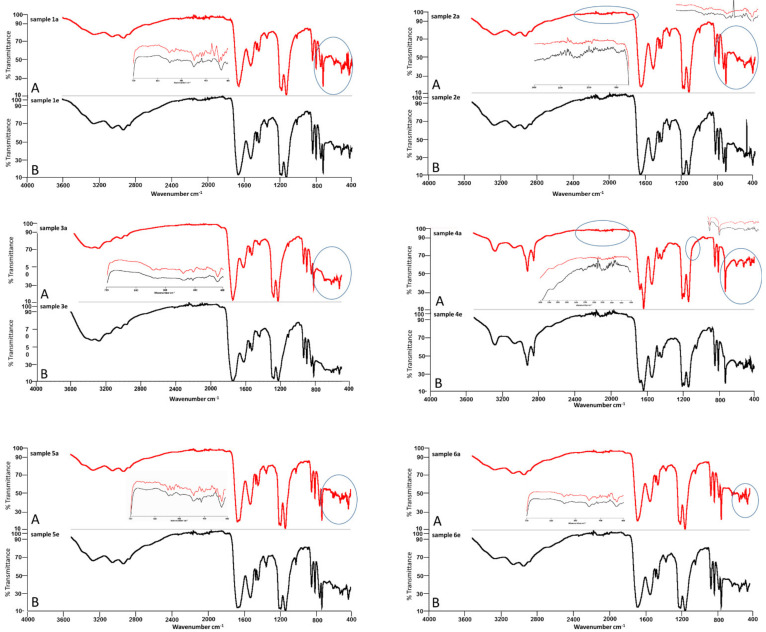
FTIR spectra of dried peptides 1–6, unexposed (**A**, samples a) and irradiated for twenty four hours (**B**, samples e). Sample numbers correspond with peptide numbers: peptide 1 is shown in samples 1a and 1e, peptide two in samples 2a and 2e, peptide three in samples 3a and 3e, peptide four in samples 4a and 4e, peptide five in samples 5a and 5e, and peptide six in sample 6a and 6e. The circles in the spectra represent the data range that changed during exposure.

The FTIR spectra of unexposed, dry peptide samples were marked on the plots with the letter “A” and irradiated for 24 h. as “B” (Figure 1). The following absorption bands were present in all FTIR spectra of the assessed peptides: 3281–3253 cm^−1^, reflecting N–H stretching vibrations that provide hydrogen bonding information in peptides and proteins; 2940–2850 cm^−1^, reflecting the stretch (-CH) of CH_2_ and CH_3_ groups in aliphatic chains; 1650–1670 cm^−1^, corresponding to amide I (C = O) bonds; and 1522–1540 cm^−1^, indicating the presence of amide II (N–H) bonds.

The spectrum of peptide one was altered in the range of 800–400 cm^−1^, a region specific to aliphatic quinines and carboxylic acid salts. In the case of peptide two, irradiation resulted in a modification of the bands at 2400–1700 cm^−1^ and below 700 cm^−1^. The differences in intensity and width of signals in the spectra of the unexposed and exposed samples of peptide three were mainly observed below 700 cm^−1^. In the basic spectrum of peptide four there were two specific signals, 1629 and 1536 cm^−1^, which differed from the spectra of peptides 1–3. The irradiated spectrum of peptide four exhibited jagged bands at 2700–1800 cm^−1^. Additionally, new signals were observed at 1044, 739, and 500 cm^−1^, and, pronouncedly, at 879 cm^−1^. The spectrum of peptide five exhibited a pattern similar to peptide four in terms of signal pairs, here observed at 1643 and 1519 cm^−1^. The modifications of the spectrum in the exposed material of peptide five were below 700 cm^−1^. The signals observed at 598, 588, and 579 cm^−1^ were weakened, whereas the signals at 481, 471, 444 cm^−1^ disappeared. In the case of the irradiated sample of peptide six, the signals at 583, 476, 450, and 433 cm^−1^ disappeared, while the signals at 566 and 491 cm^−1^ shifted towards shorter wavelengths.

Gathered data from the FTIR experiments for the evaluated peptides: non-radiated and after irradiation are shown in Table 3.

### 2.3. Antioxidant Properties of Evaluated Peptides

**Figure 2:**
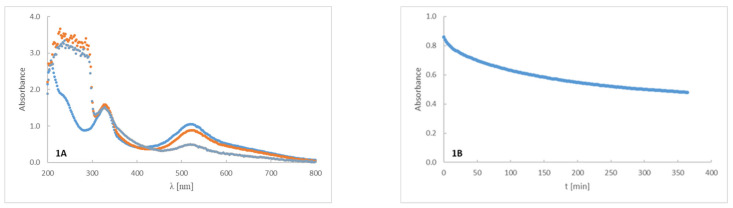

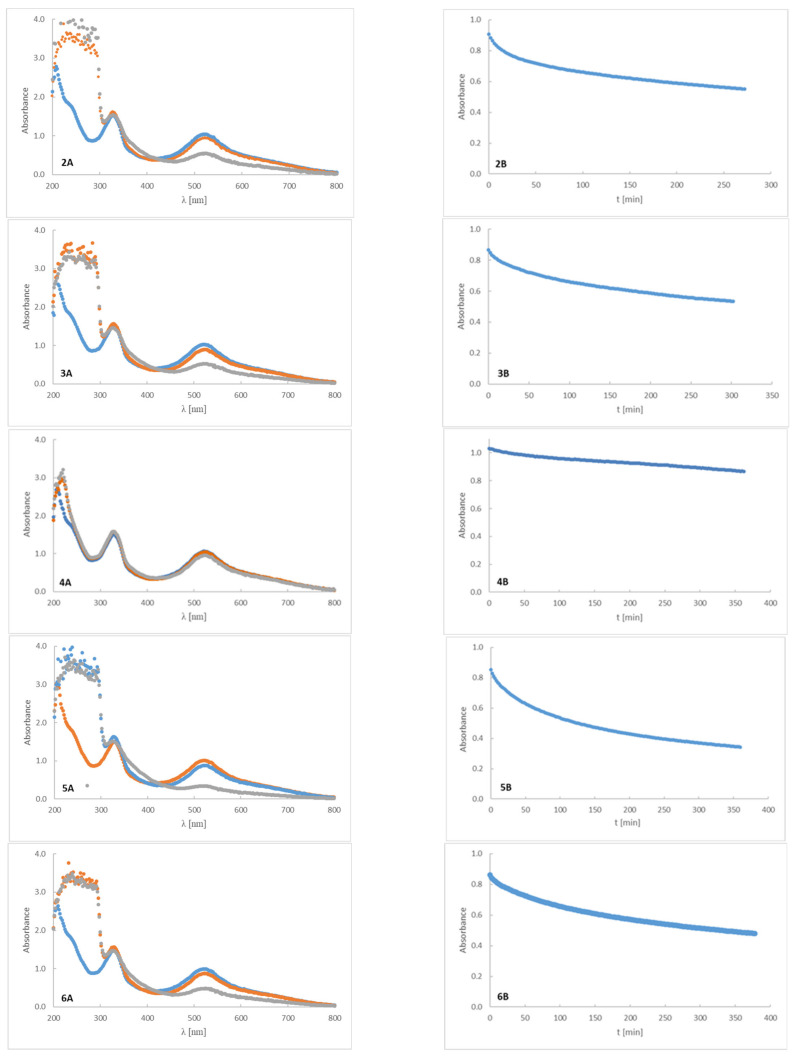
The DPPH radical spectra (**-**) and the spectra of the DPPH solution doped with respective peptide solutions at time zero (**-**) and after 6 h (**-**). Left panel, plots 1A–6A: the degradation of DPPH radicals resulting from the reaction with a test peptide (**-**); right panel, plots 1B–6B: absorbance decrease was measured at 522 nm for peptide samples 1–6.

The spectra of DPPH radicals (blue lines), the spectra of DPPH radicals for peptide at time zero (orange lines), and the spectra of DPPH radicals for peptide measured after six hours (grey lines) are presented on plots 1A–6A of Figure 2. During the experiment, no additional signals appeared that could indicate the formation of additional reaction products. DPPH absorption bands were observed at 332 nm and 522 nm. At 522 nm, no overlapping signal was observed. Signals resulting from the mixture of DPPH and peptide (Figure 2, plot 4A), ranging from 210 to 290 nm, were narrower than those shown in graphs 1A—6A, presumably due to the lack of tryptophan in compound four. A decrease in DPPH radical absorption over time, when reacted with 2 mg/mL of the selected peptide, is demonstrated on plots 1B-6B of Figure 2. The reaction had two stages: in the initial 50 min, a rapid decrease of DPPH radical absorption over time was observed during its reaction with peptides, followed by a slower decrease. Plot 4B of Figure 2 presents the smallest decrease in absorbance with time; the reaction of P4 with DPPH was the slowest, and showed a high fitness to a linear function.

**Figure 3:**
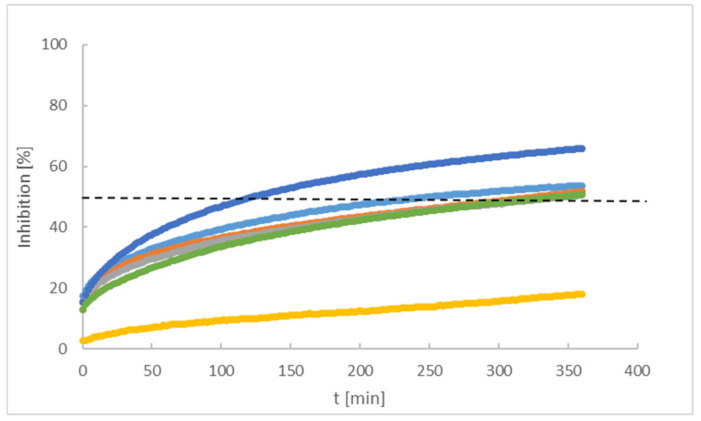
Percentage of DPPH inhibition by various peptides: P1 (**-**), P2 (**-**), P3 (**-**), P4 (**-**), P5 (**-**), P6 (**-**).

The antioxidant properties of the tested peptides are presented as a percentage of the DPPH radical inhibition with time, shown in Figure 3. The comparison of peptides 1–6 (2 mg/mL), in terms of time required for the extinction of fifty percent of the DPPH (black dotted line in Figure 3), indicates a high activity of compound five (124 min) and the remaining are ordered as P1, P2, P6, and P3. Peptide four inhibited less than approx. 20% of radicals over six hours.

Statistical analysis of the data presented in Figure 3 was performed using a normal distribution. In the zero hypothesis we assumed that t_50,_ for each assessed peptide, was the same. Rejection of the zero hypothesis was set at a significance level 0.005; results in I type errors are shown in Table S1.

### 2.4. Antimicrobial Activity of the Peptides Evaluated in Planktonic Colonies

**Figure 4:**
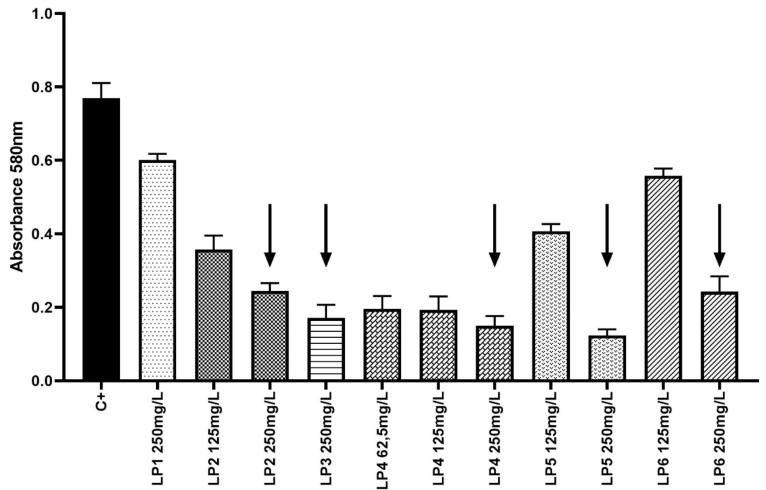
Survival of planktonic *S. aureus* cells exposed to the specific concentrations of lipopeptides P1–6. C+: control setting of microorganism growth with no peptide applied, considered 100% of potential growth. Black arrows indicate MBC—minimal bactericidal concentrations of peptides, confirmed by plate culturing.

The MBC values of P2–P6 were equal to MIC (250 mg/L). No MBC value was observed for P1. Survival data for *S. aureus* plankton cells exposed to the peptides are presented in Figure 4.

### 2.5. Assessment of Peptides Activity against Staphylococcus aureus Biofilm

**Figure 5:**
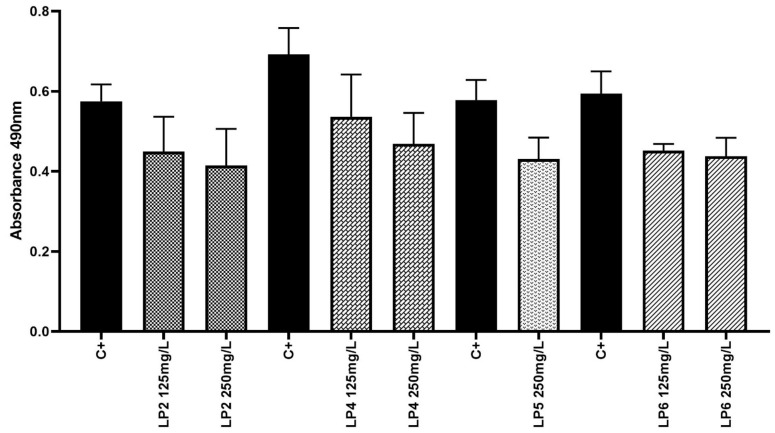
Survival of *S. aureus* in biofilm exposed to P2,4,5,6. C+ represents microorganism growth in the absence of peptides (control). The differences between control and lipopeptide-treated cultures are statistically significant (ANOVA test with Tukey’s multiple comparisons test, *p* < 0.05).

MBEC values were not observed for the tested peptides P1–P6. Nevertheless, the peptides P2, P4, P5, and P6 exhibited significant reduction of staphylococcal biofilm. The reduction was observed after exposure to 125 and 250 mg/L of P2, P4, and P6, as well as exposure to 250 mg/l of P5, confirmed by ANOVAs with Tukey’s multiple comparisons test, *p* < 0.05 (Figure 5).

### 2.6. Safety Profile of Evaluated Peptides Assessed in the Fibroblasts

Having proven the activity of tested peptides against staphylococcal planktonic cells and biofilm, in the next step of investigation we analyzed potential cytotoxic effects of peptides against eukaryotic fibroblast cell line L929.

**Figure 6:**
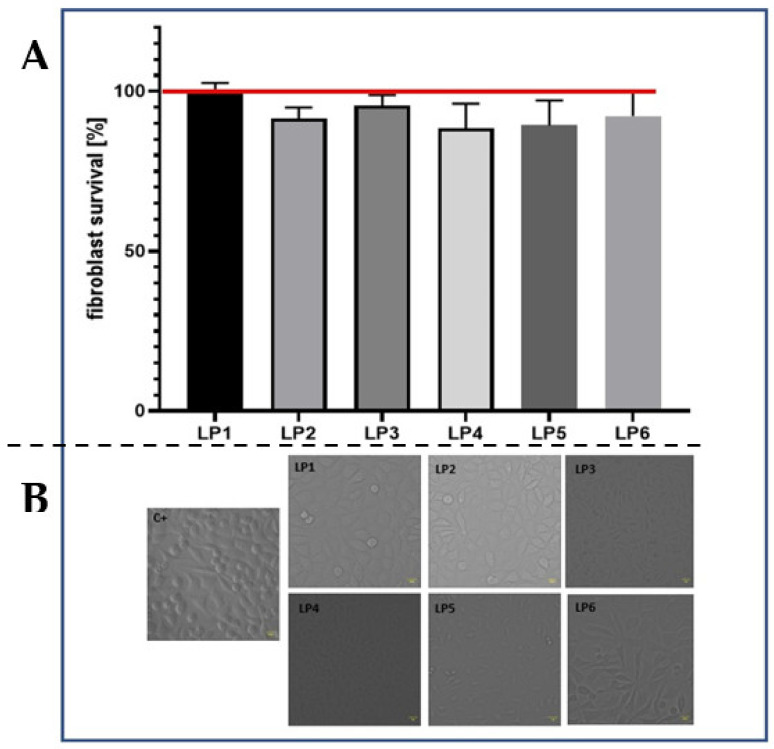
Evaluation of the cytotoxicity of P1–P6. (**A**): Survival of fibroblasts [%] exposed to P1–P6 in concentration equal to 250 mg/L. The red line indicates the growth level of fibroblasts unexposed to P1–P6 (growth control); (**B**): intact fibroblast morphology after exposure to P1–P6. LP1–LP6 denominate P1–P6.

Results, presented in Figure 6, indicate that an applied concentration of peptides did not result in significant decrease in fibroblast survival (Figure 6A) nor modifications in the cells’ morphology (Figure 6B). A cytotoxic effect, defined either as a decrease greater than 30% of a cell’s viability or a morphological change, was not observed in the presence of peptides P1–P6.

### 2.7. Antimicrobial Activity of the Peptides Released from Paper Disks and Specific BC Cellulosic Carriers

**Figure 7:**
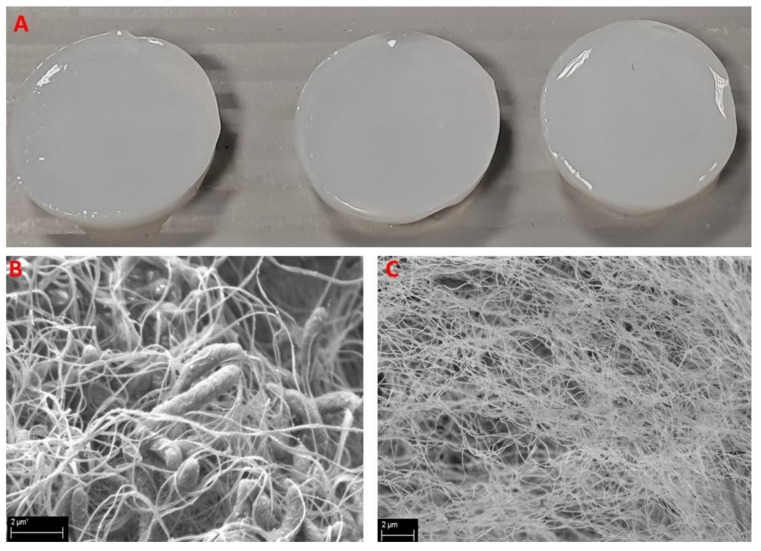
Bacterial Cellulose Carrier presentation. (**A**): Macrophotography of BC carriers applied to absorp peptides; (**B**): BC carriers during the process of formation by *K. xylinus* bacteria (rod-like shapes); (**C**): BC carrier after bacterial removal reveals its porous structure. SEM—Zeiss EVO MA 60, magn. 50,000 × (**B**) and 25,000 × (**C**).

The average wet weight of produced BC carriers was 818.3 ± 31.8 mg, while the dry weight was 13 ± 3.9 mg, and the resulting water content of BC carriers was approximately 98.5% of the total weight. An example of BC carriers used in these experiments, and a SEM visualization of BC’s porous structure, are presented in Figure 7.

**Figure 8:**
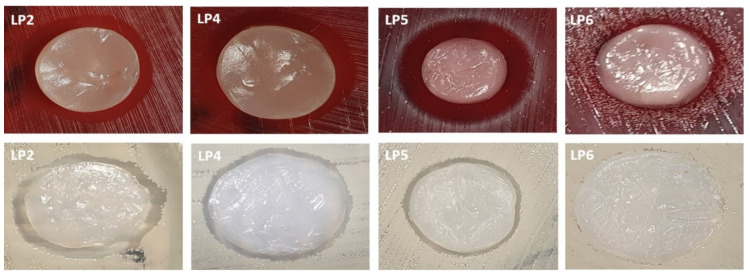
Inhibition zones resulting from the antimicrobial activity of P2, P4, P5, and P6 released from BC carriers, against *C. acnes* (upper panels) and against *S. aureus* (lower panels). LP1–LP6 denominate P1–P6.

The inhibition zones of bacterial growth after exposure to peptides released from applied BC carriers are presented in Table 4 and Figure 8.

## 3. Discussion

The search for new drugs against skin infection is an ongoing and developing process; there is a necessity to improve existing therapies or else evaluate new therapeutic countermeasures [27]. The desired directions for improvement include antimicrobial activity and decreased cytotoxicity—preferably, a lack of cytotoxic effects in new peptides aimed toward topical application. Such peptides should also display antioxidant properties and be resistant to solar radiation [28]. The peptides evaluated in the present work consisted of various amino acids: (WK)_2_-KWK-NH_2_ (P1), (WKWK)_2_-KWKWK-NH_2_ (P2), (WR)_2_-KWR-NH_2_ (P3), (C12)_2_-KKKK-NH_2_ (P4), (KWK)_2_-KWWW-NH_2_ (P5), (KK)_2_-KWWW-NH_2_ (P6). Their differentiated structures resulted in differentiated antioxidant, antimicrobial, and toxicological properties (Figure 2, Figure 3, Figure 4, Figure 5 and Figure 6). Similarly, the stability assessed after light-radiation in the range of 200–1100 nm depended upon the structure of the irradiated peptide [29,30].

In our FTIR experiments, samples P1–P6 were sensitive to 24 h of solar irradiation. The polypeptide backbone of P1–6 is composed of nine amide bands related to the vibration in CONH groups of each protein chain’s [31] secondary structures. IR may also provide information on protein structural stability and dynamics. Comparing the spectra of samples P1–P6 unexposed to solar irradiation, there was a 746 cm^−1^ signal associated with the bending vibrations of the CH bonds in the indole ring and with deformations of the entire indole skeleton [32]. The signal was not present in the FTIR spectrum of P4 as it does not contain tryptophan. The irradiated P1–P6 exhibited deformation vibrations of O = CN groups (amide bands IV, 625–767 cm^−1^), deformation vibrations of NH groups extending beyond the plane (amide bands V, 640–800 cm^−1^), and deformation vibrations of C = O groups extending out of the plane (amide VI bands, 537–606 cm^−1^) [33]. The irradiated P2 and P4 additionally presented changes in the area of ca. 2700–1700 cm^−1^ during irradiation, whereas change in the range of 2500–3300 cm^−1^ was attributed to the vibrational stretching of the acid group O-H (bond-forming OH hydrogen group), and the broad band of 2000–1500 cm^−1^ corresponded mainly to the vibrational stretching of double bonds (C = O, C = C, C = N). Data from the literature confirm that solar energy results in conformational modification of collagen molecules [34], however spectroscopic results, similar to those performed in our experiments on samples P1–P6, cannot confirm destruction of the chain.

**Figure 9:**
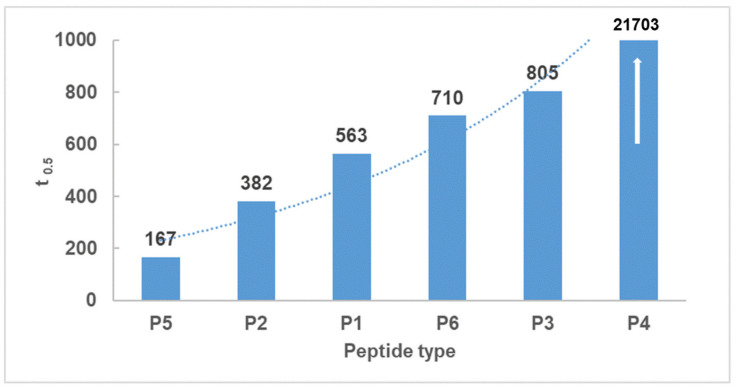
The variability of radical concentration half-lives (t_0.5_) reacting with individual peptides. The white arrow indicates that t_0.5_ for P4 is exceeds the range of the y-axis.

The compound P5 presented the highest antioxidant properties, and was followed by P1, P2, P6, P3, and P4 respectively. The antioxidant properties of the tested compounds did not depend on the molar concentrations, and the half-life times of radical concentration (t_0.5_) for P1–P6 at 0.0008 mol/L presented different values (Figure 9).

The antioxidative activity of peptides is related to amino acid composition, structure, and sequences [35,36]. In specific experimental conditions some amino acids, such as glycine, methionine, or tryptophan, have been reported to accelerate oxidation [37], in contrary to their described antioxidant properties. There is a relationship between the value of the average hydrophobicity of a peptide and its antioxidant activity [38]. It is also well-acknowledged that peptides containing alkali amino acid residues, such as histidine and lysine, possess highly antioxidant activities [39]. Antioxidant structures, as electron donors, may be evaluated in respective calculations of the energies in their molecular orbitals; this is similarly to estimating the prooxidative properties of prooxidants, which are electron-acceptors, [40]. The peptides evaluated in our study consisted of polar and non-polar amino acids in specific proportions. The equal numbers of polar lysine residues and non-polar tryptophan residues in P5 resulted in the highest antioxidant activity. In peptides one and two, which also displayed high antioxidant activity (ranked second and third, respectively) there was one more lysine than tryptophan residue; the number of lysine residues in the molecule exceeded the number of tryptophan residues by one. In turn peptide six, in which the ratio of lysine to tryptophan residues was 5:3, ranked fourth in terms of antioxidant properties. This pattern did not hold for P3 and P4, which differed in composition compared with the abovementioned; P3 contained arginine, and P4 did not contain aromatic rings. The absence of aromatic amino acids residues may have contributed to the decreased antioxidant activity of P4, which was confirmed in the case of peptides with large side groups, such as histidine with an imidazole group, or tryptophan with an indolic group [41].

P2 and P4–6 displayed recordable antimicrobial activity (Figure 5). Results of performed MBC analyses (Figure 4) revealed that peptide four, containing, among other elements, four lysine residues in the form of polylysine (KKKK), and displayed the highest anti-staphylococcal activity among those tested. The MBEC value was recorded at 250 mg/L of P4, but strong reduction of staphylococcal cell number was also observed after exposure to 62.5 mg/L of this peptide. Peptide two ranked second with regard to antimicrobial activity, and contained seven lysine residues; however, contrary to P4, it was intertwined with tryptophan residues. Interestingly, P5 and P6, with five lysine residues each, were able to eliminate staphylococcal cells in concentrations of 250 mg/L and to reduce their number in concentrations of 125 mg/L, while P1, with four lysine molecules, displayed a lack of antimicrobial activity (Figure 4). Thus, it may be hypothesized that both the number of lysine residues, as well as their localization within a peptide are of great importance with regard to its antimicrobial activity. Notably, these considerations remain congruent with data presented by other researchers that show positive correlation between the antimicrobial potential of peptides with increasing number of lysine residues, especially if they occur in a poly form [42]. Contrary to P1,2,4,5, and 6, P3 contained only one residue of lysine. Moreover, P3 contained arginine residues, which were absent in other evaluated peptides. This peptide also displayed recordable antimicrobial activity, however only in the highest concentrations of 250 mg/L. This may be explained in a fashion similar to the case discussed of lysine-containing peptides. Additionally, arginine’s activity is improved versus microorganisms when it occurs in the form of polyarginine, while analysis of peptide five found it to be intertwined with tryptophan. It should be noted that P1–P3, P5, and P6 contained tryptophan residues, which present high affinity to the interfacial regions of biological membranes. Therefore, tryptophan may be considered an anchoring molecule, binding peptides to microbial cells. P2 contained the highest number of tryptophan residues (6) out of all peptides tested and, as it has already been stated, it ranked second in antimicrobial activity. On the other hand, P4, which contained no tryptophan, ranked first. The superior activity of P4 may result from the non-restricted access of peptides to bacteria in the experimental setting. In the human body, the microorganisms are usually deposited within the tissues and body fluids, which increases the importance of the anchoring properties of tryptophan [43].

P2 and P4 presented the highest antibiofilm activity (Figure 5), however the MBEC value was not achieved. This finding agrees with the generally recognized protective function of bacterial biofilms, which allows to exceed tolerances up to 1000 times comparing to their bacterial planktonic counter-parts [44]. Nevertheless, the analyses presented in Figure 4 were performed for standard, immersed biofilm. One should observe the well-recognized fact that antimicrobial peptides act more efficiently against Gram-negative than Gram-positive (scrutinized in this study) pathogens [45]. This phenomenon is related to the interaction of peptidoglycan and teichoic acids present among Gram-positive bacteria, which may act as a trap for peptides. This satisfactorily explains the relatively high concentrations needed to reach MIC value or a reduction of biofilm (Figure 4 and Figure 5). Nevertheless, the concentration of antimicrobial substances in modern antiseptics used for local treatment of wound and/or skin lesions frequently exceeds a value of 250 mg/L. The most prominent examples are octenidine- and polihexanide-containing antiseptics, of the aforementioned concentrations of antimicrobial substances equal of 1000 mg/L [46,47]. Regardless of the numerous positive features of commercial applied antiseptics for wounds, they nonetheless display specific limitations that peptides do not. The application of a common antiseptic agent, chlorhexidine, may cause accidental or recurrent inflammatory and allergic reactions [48], while the use of polihexanide may display toxic effects to keratinocytes and lead to dermatitis in patients [49], and wound irrigation with octenidine dihydrochloride may result in severe complications, such as aseptic necrosis and chronic inflammation in penetrating hand wounds [50]. No such side effects were reported as a result of the studied peptide’s activities, and, additionally, increased microbial tolerance/reduced susceptibility towards chlorhexidine [51], octenidine [52] and polihexanide [53] has already been indicated.

On the other hand, due to an unspecific mode of action, the application of antimicrobial peptides against Gram-positive pathogens is a subject of broad investigation to numerous research teams [54].

The application of specific-carrier BC, presumed for topical use, was evaluated in combination with P2 and P4–P6 to reflect the conditions of a cutaneous formulation with antimicrobial drugs. The antimicrobial activity of the abovementioned peptides, absorbed into the BC, against *S. aureus* and *C. acnes* was assessed. P2, having the highest positive charge of six, demonstrated remarkable antimicrobial activity against both bacteria. It should be noted that the applied concentrations of peptide (1000 mg/L) was the same as concentrations of octenidine- or polyhexanide-based antiseptics, applied globally, for skin and wound treatment. P5 was highly active against *C. acnes*, and its antioxidant properties were also superior. This combined activity may be of special importance, as *C. acnes* colonization leads to the formation of reactive oxygen species, including peroxide anions, which may form peroxynitrates that are responsible for the breakdown of keratinocytes [55]. This phenomenon goes in hand with the observation that endogenous, host -defense peptides [56,57] also have positive net charge and amphiphilicity, which are responsible for their antimicrobial activity. The performed assays confirmed a lack of cytotoxicity of P1–P6 against fibroblast lineages, which not only enhanced the applicative potential of synthetized peptides on skin surfaces, but also may be regarded as a proof of concept for the possibility of an undisturbed process of healing lesions in vivo, which is mediated by fibroblasts.

## 4. Materials and Methods

### 4.1. Synthesis and Characterization of the Peptides

#### 4.1.1. Peptides Synthesis Preparation

Rink amide AM resin and the amino acids Fmoc-Lys (Boc)-OH, Fmoc-Lys (Fmoc)-OH, Fmoc-Arg (Pbf)-OH, and Fmoc-Trp (Boc)-OH were obtained from Iris Biotech (Marktredwitz, Germany). Dodecanoic acid, coupling reagents, and solvents such as N,N-dimethyl formamide (DMF), dichloromethane (DCM), 1-hydroxybenzotriazole (HOBt), trifluoroacetic acid (TFA), and acetonitrile (ACN) were obtained from from Merck (Darmstadt, Germany).

Peptide sequences were de novo designed to present positive charge by the incorporation of arginine or lysine residues. Tryptophan residues and dodecanoic fatty acid were used to facilitate insertion into bacterial membranes. Peptide compounds were manually synthesized by Fmoc solid-phase peptide synthesis using Rink amide AM resin (100–200 mesh; loading 0.48 mmol/g). The coupling reaction of the amino acids was made with the activators DIC and HOBt, with three times the molar excess of each amino acid and activator, and dissolved in DMF/DCM (1:1; *v/v*) mixture. Deprotection was carried out with 20% (*v/v*) of piperidine in DMF. Deanchoring of the peptides from the resin was achieved with a TFA/TIS/H2O mixture in a volume ratio (95:2.5:2.5).

#### 4.1.2. Peptides Purity and Structure

Purity of the peptides was analyzed by reverse-phase high-performance liquid chromatography (RP-HPLC) in a Shimadzu Nexera chromatograph with a DAD detector at 214 nm fitted with Eurospher (100 × 4.6 mm) columns (Knauer, Berlin, Germany) using ACN:TFA (0.1%) and H2O:TFA (0.1%) as the mobile phase. The identity of each peptide was verified by matrix-assisted laser desorption time-of-flight (MALDI-TOF) spectrometry on MALDI-TOF/TOF 5800 (Sciex, Illinois, USA). The peptides whose identities were confirmed via MS spectra were freeze-dried (Christ, Hannover, Germany) and stored as dry powder at −20 °C.

#### 4.1.3. FTIR Spectroscopic Studies

FTIR measurements were performed using a Thermo Scientific Nicolet iS50 FT-IR Spectrometer with an attenuated total reflectance (ATR) device (Thermo Fisher Scientific, Waltham, MA, USA). Samples of the non-irradiated peptides 1–6 were evaluated at the beginning of the experiment, and after every six hours the during 24 h of the irradiation process, presented below. The samples were irradiated with a home-built system consisting of a high-pressure mercury lamp HBO 200DC of 200 W (Osram, Munich, Germany), and cuvette-enabling safe sample processing [57]. The measurements were carried out at a constant temperature, maintained in the irradiation system.

#### 4.1.4. DPPH (2,2-diphenyl-1-picrylhydrazyl) Radical Scavenging Activity

The DPPH spectrometric method described by Brand, Williams et al. [58,59] was applied due to its efficiency on lipophilic and hydrophilic samples [60]. The evaluation of free radical scavenging capacity (FRSC) was based on DPPH (Merck, Darmstadt, Germany) absorbance intensity resulting from its reaction with surfactants. A PG Instruments UV–Vis T60 spectrophotometer (Alab, Warszawa, Poland), interfaced with a computer with a quartz cuvette and of path length 1.0 cm at 25 °C, using a total volume of 3.0 mL, was applied in the range 190–900 nm. As a sample (As) 1.0 mL of P1–P6 aqueous solution (2 mg/mL) and 2.0 mL of ethanolic DPPH solution (5.9·10–2 mg/mL) were mixed and evaluated. As a blank (Ab), 1.0 mL of water, mixed with 2.0 mL of ethanol, was used. Absorbance decrease at 522 nm was measured in 2 min intervals for 6 h. FRSC was calculated as a percentage with the following equation: FRSC (%) = [(Ab – As)/Ab] × 100%. All measurements were performed in triplicate.

### 4.2. Peptide Antimicrobial and Cytotoxic Properties

#### 4.2.1. Bacterial Strains and Eukaryotic Cell Lines

For experimental purposes, the American type culture collection’s strains of *S. aureus* 6538 and *C. acnes* 6919 were applied. To evaluate the cytotoxic potential of the tested peptides, fibroblasts L929 (ATCC^®^ CCL-1) were used.

#### 4.2.2. Assessment of the Minimal Inhibitory Concentration of Peptides against *Staphylococcus aureus*

A loop of staphylococcal culture grown on Columbia agar plate (BioMaxima, Poland) was transferred to liquid tryptone soya broth (TSB, BTL) and incubated at 37 °C for 24 h. Next, culture’s optical density of 1 McFarland (3 × 10^8^ cfu/mL) was established using a densitometer (Biomerieux, Poland). Subsequently, the suspension was diluted in the Miller-Hinton Broth (M-H, BioMaxima, Poland) to reach the density of 1 × 10^5^ cells/mL. Afterwards, serial dilutions of each peptide was performed in M-H Broth in 96-well plate (BioStar, Germany). Next 100 μL of previously-prepared microbial suspension was added to each well. The highest concentration of each peptide tested in this experimental setting was thus 250 mg/L and it dropped twice in each subsequent well of 96-well plate up to 0.48 mg/L. Plates were incubated for 24 h/37 °C in shaker (Lab Companion, JeioTech, Daejeon, Korea).

After incubation, the turbidity of bacterial culture was measured using Thermo Scientific Multiskan Go spectrometer (Thermo-Fischer Scientific, Vantaa, Finland) with wavelength of 580 nm. The microbial culture where no peptide was added served as growth control. The sterility control setting was M-H broth with no microorganism added. The tetrazolium salt (TTC) test served as additional analysis of microbial cell number reduction. All experiments were performed in triplicate.

#### 4.2.3. Assessment of Minimal Bactericidal Concentration of Peptides against *Staphylococcus aureus*

The following standard procedure of MBC evaluation according to CLSI (Methods for Determining Bactericidal Activity of Antimicrobial Agents; Approved Guideline, document M26A, Global Laboratory Standards for a Healthier World, https://clsi.org, accessed on 15 December 2020) was performed. The 96-well plates with bacteria and peptide concentrations were performed and incubated as it was described in Section 2 of Material and Methods. Then the M-H agar plates with spots containing staphylococcal culture were incubated at 37 °C for 24 h. Lack of colony presence in the spot after incubation was considered as lack of microorganism growth, while presence of microbial colony in the spot was considered presence of microorganism growth. All experiments were performed in triplicate.

#### 4.2.4. Assessment of Peptides Activity against *Staphylococcus aureus* Biofilm

A total of 100µL of *S. aureus* suspension (10^5^ cfu/mL) in TSB) medium was introduced to wells of 96-well plate and incubated at 37 °C/24 h without shaking. Next, whole medium was removed, leaving biofilm-forming organisms attached to the bottom of a 96-well plate. Subsequently, geometric dilutions of peptides in TSB (62.5–250 mg/L) were applied to the wells and left for another 24 h at 37 °C. The culture with no added peptide served as a positive control for microorganism growth, while the well containing the sterile TSB only served as a sterility control for the experiment. After incubation, 5 µL of triphenyl tetrazolium chloride (TTC, Sigma Aldrich, Taufkirchern, Germany) was added to each well and incubated for 5 h at 37 °C. A change of colorless TTC to red formazan confirmed the presence of metabolically active microorganisms. Next, the formazan was dissolved in methanol and its concentration was measured using a Thermo Scientific Multiskan Go spectrometer with a wavelength of 490 nm. In parallel, biofilms in plate wells were agitated using an automated pipette, removed from 96-well plates, and spotted on a stable (M-H) agar plate (Biomaxima, Lublin, Poland) where they were incubated for 48 h. The presence of living colonies on agar indicated the lack of MBEC, while the absence of colonies in the place where spotting was performed confirmed that the particular concentration of peptide can be considered the MBEC.

#### 4.2.5. Assessment of Peptides Cytotoxicity towards Eukaryotic Fibroblast Line

Reagents: Dulbecco’s modified eagle’s medium DMEM; Dulbecco’s phosphate buffered saline DPBS; trypsin-EDTA; fetal bovine serum FBS; penicillin with streptomycin and amphotericin B were purchased from Biowest, France. MTT ((3-(4,5-dimethyl-2-thiazolyl)-2,5-diphenyl-2H-tetrazolium bromide) (M5655) was obtained from Sigma Aldrich, Germany. To prepare MTT work solution, 5 g/L (*w/v*) of MTT was dissolved in PBS and diluted 10 times in DMEM to final concentration 0.5 g/L (*v/v*). The 1 mg of each peptide was introduced to 1 ml of DMEM medium for fibroblast culturing. Samples were incubated 24 h/37 °C. 100 µL of cell suspension (cell density 1.5 × 105 mL) was seeded to wells in 96-wells plate (VWR, USA) and cultured overnight in incubator 37 °C and 5% CO2 Binder C 150 UL CO2 incubator (Binder, Germany). When the confluency was reached, medium was carefully removed and 100 µL of DMEM was added to the wells (200 µL to positive control). 100 µL of previously prepared substances was poured to the wells. The plate was incubated overnight at 37 °C with 5% CO2. Then, the growing medium was removed and 100 µL of MTT work solution was added to the cells and incubated for 2 h at 37 °C with 5% CO2. Next, the solution was carefully removed. Having plates dried (10 min, 37 °C), 100 µL of isopropanol was added to the wells. The plate was shaken for 30 min at 400 rpm Plate Shaker-Thermostat PST-60HL-4 (Biosan, Latvia). Color intensity was measured at wave lengths λ = 570 nm and λ = 630 nm using spectrometer Multiscan^®^ GO (Thermo Scientific, USA).

### 4.3. Evaluation of the Antimicrobial Activity of the Peptides Dposited on the Bacterial Cellulose (BC)

#### 4.3.1. Culturing and Preparation of BC and Peptides Absorption into BC

To produce BC, *K. xylinus* ATCC 53524 was used. The strain was cultivated in stationary conditions for 7 days at 28 °C in 24-well using a Hestrin-Schramm (H-S) medium. Obtained BC carriers were rinsed with water. Next, to remove bacterial cells and media components, the BC carriers were purified in 0.1 M NaOH (POCH, Poland) for 90 min at 80 °C. After purification, BC disks were immersed in distilled water and incubated with shaking. The efficiency of BC purification was performed using Scanning Electron Microscope ZEISS EVO MA as we described it elsewhere [61]. The pH value was measured every 3 h. The washing procedure was continued until there was no change in pH. To calculate water content of BC, the carrier was dried at 37 °C. Every day the weight was assessed using electronic balance PA114CM/1 (Ohaus, Germany) until no further drop of weight was observed. The wet BC carriers were transferred to 24-well plate and immersed with 800 µL of 2 mg/mL of peptide. The plate was left for 24 h/4 °C. The control setting of this experiment was BC carrier absorbed with octenidine dihydrochloride (antiseptic substance of confirmed antimicrobial activity) and 0.9% NaCl, introduced to the BC carrier. Additionally, as carrier’s control setting, the peptides were introduced to paper discs (Whatmann, USA) of 6 mm diameter (used for conventional antibiotic therapy).

#### 4.3.2. Assessment of *S. aureus* and *C. acnes* Growth Inhibition Zones after Exposure to Peptides Absorbed into BC

Paper discs and BC carriers absorbed with peptides were placed onto the surface of the appropriate agar medium seeded with the suspension of *S. aureus* or *C. acnes* at a density of 0.5 McFarland (c.a. 1.5 × 10^8^ cfu/mL) using a densitometer (Biomerieux, Poland). Next, agar plates were carried out at 37 °C for 24 h. The results were presented as a growth inhibition zone diameter, expressed in mm. The diameters of BC carriers and paper discs were subtracted from the diameters of growth inhibition zones. The tests were performed in triplicates.

### 4.4. Statistical Analysis

Calculations were performed using the GraphPad Prism version 7 software (GraphPad Co., San Diego, CA, USA). The normality of distribution was assessed by means of the D’Agostino–Pearson omnibus test. Because all values were non-normally distributed, ANOVA tests with Tukey’s multiple comparisons were applied. The results of these statistical analyses were considered significant if they produced *p*-values < 0.05.

A normal distribution was used in compiling the data on antioxidant properties in Section 2.3.

## 5. Conclusions

The P5 compound presented the highest antioxidant properties, and was followed by P1, P2, P6, P3, and P4 respectively. The antioxidant properties of the tested compounds were influenced by amino acid residues such as tryptophan, lysine, and arginine in the peptide structure, which increased the antioxidant properties of the compound. The potential instability after extensive exposure to sunlight may influence the application possibilities of the assessed peptides. Cytotoxic effects were not observed in the presence of the evaluated peptides, which affirms the possibility of future topical applications. The MBC values for P2–P6 against *S. aureus* were 250 mg/L, while MIC and MBC values werenot observed for P1, suggesting the possibility of anti-infective applications for P2–P6 on skin; however, only P2 and P4 were characterized by remarkable antimicrobial activity against both *S. aureus* and *C. acnes*, when applied in the form of molecules absorbed by BC. Application of the selected peptides P2, P4, P5 absorbed into BC resulted in antimicrobial activity, and presumably provides direction for further research on BC as an effective carrier for antimicrobial peptides. The obtained data may enhance the development of efficient anti-acne pharmaceutical preparations.

## Figures and Tables

**Table 1:** Physicochemical properties of the tested peptide compounds.

Compound Number	Structure	Net Charge	[M + H]^+^	MWcalc.
P1	(WK)_2_-KWK-NH_2_	+5	1088.53	1087.64
P2	(WKWK)_2_-KWKWK-NH_2_	+8	2031.17	2030.16
P3	(WR)_2_-KWR-NH_2_	+5	1172.67	1171.66
P4	(C_12_)_2_-KKKK-NH_2_	+3	894.7	893.7
P5	(KWK)_2_-KWWW-NH_2_	+4	1588.90	1587.89
P6	(KK)_2_-KWWW-NH_2_	+4	1216.74	1215.73

W—tryptophan; K—lysine; R—arginine; C12—dodecanoic acid.

**Table 2:** Structures of tested peptides P1–P6. P1–P6 are the abbreviations of the evaluated peptides, described in the text.

P1 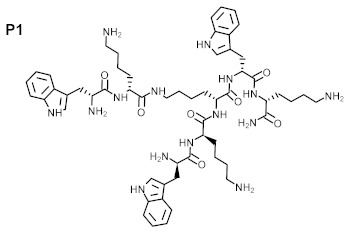	P2 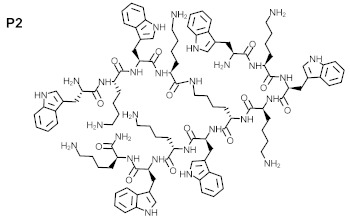
P3 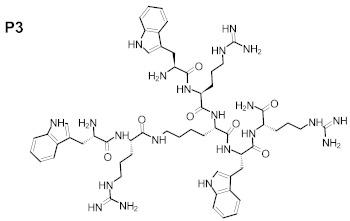	P4 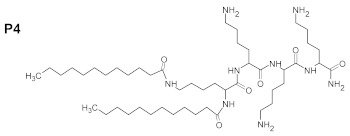
P5 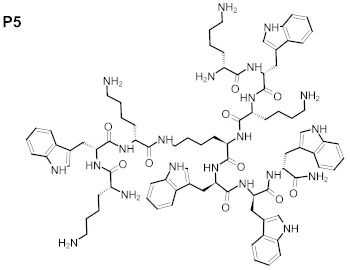	P6 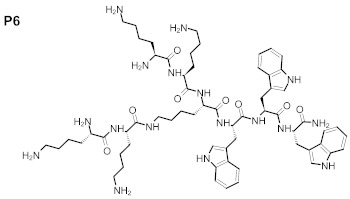

**Table 3:** The specific and altered signals of evaluated peptides 1–6.

Type of Peptide	Specific Bands [cm^−1^]	Bands Alteration Regions Observed after Irradiation [cm^−1^]
P1	3253, 2934, 1654, 1522	800–400
P2	3273, 2932, 1654, 1522	2400–1700, <700
P3	3277, 2940, 1651, 1529	<700
P4	3281, 2919, 1671, 1629, 1536	2700–1800,1044, 879, 739, 500
P5	3274, 2935, 1663,1643, 1519	<700 cm, 598, 588, 579, 481, 471, 444
P6	3265, 2938, 1663, 1522	583, 566, 491, 476, 450, 433

**Table 4:** Inhibition zones of *S. aureus* and *C. acnes* growth as a result of activity of peptides P2, P4, P5 and P6 released from paper discs and BC carriers.

	Paper Discs				BC Carriers			
	Inhibition Zone [mm]				Inhibition Zone [mm]			
Compound Name	P2	P4	P5	P6	P2	P4	P5	P6
*S. aureus*	0	0	1	0	4.6 ± 0.5	4.6 ± 0.5	3 ± 0	0
*C. acnes*	1.6 ± 0.9	0	2	0	4.8 ± 0.3	3.6 ± 0.5	5.6 ± 0.6	1 ± 0

## Data Availability

Not applicable.

## Supplementary Materials

The following are available online at https://www.mdpi.com/article/10.3390/ijms22147466/s1, Table S1. The *p*-value of type I error.
