# Supplementary material for: Antimicrobial and Antioxidative Activity of Newly Synthesized Peptides Absorbed into Bacterial Cellulose Carrier against Acne vulgaris
Source: Int J Mol Sci. 2021 Jul 12;22(14):7466. doi: 10.3390/ijms22147466 (PMC8306634; doi:10.3390/ijms22147466)
Supplement: Supplementary file 1 [file ijms-22-07466-s001.zip › ijms-1264034-supplementary.pdf]

Supplementary Materials

Table S1. The p-value of type I error.

	**P1**	**P2**	**P3**	**P4**	**P5**	**P6**
P1	1.00000	0.00000	0.00000	0.00000	0.00000	0.00000
P2	0.00000	1.00000	0.07180	0.00000	0.00000	0.70087
P3	0.00000	0.07180	1.00000	0.00000	0.00000	0.17069
P4	0.00000	0.00000	0.00000	1.00000	0.00000	0.00000
P5	0.00000	0.00000	0.00000	0.00000	1.00000	0.00000
P6	0.00000	0.70087	0.17069	0.00000	0.00000	1.00000
